# Tilapia Skin-Derived Peptide PFRMY Attenuates Melanogenesis and Tyrosinase Activity via α-MSH/PKA/CREB Signaling Pathways in B16F10 Murine Melanoma Cells

**DOI:** 10.3390/foods15081378

**Published:** 2026-04-15

**Authors:** Yuqiong Song, Chen Lu, Shengjun Chen, Yongqiang Zhao, Hui Huang, Huan Xiang, Xiaoshan Long, Xiao Hu

**Affiliations:** 1 Key Laboratory of Aquatic Product Processing, Ministry of Agriculture and Rural Affairs, South China Sea Fisheries Research Institute, Chinese Academy of Fishery Sciences, Guangzhou 510300, China; syq1010730529@163.com (Y.S.); 17373659595@163.com (C.L.); chenshengjun@scsfri.ac.cn (S.C.); zhaoyq@scsfri.ac.cn (Y.Z.); huanghui@scsfri.ac.cn (H.H.); xianghuan@scsfri.ac.cn (H.X.); longxiaoshan@scsfri.ac.cn (X.L.); 2 Key Laboratory of Efficient Utilization and Processing of Marine Fishery Resources of Hainan Province, Sanya Tropical Fisheries Research Institute, Sanya 572426, China

**Keywords:** tilapia (*Oreochromis niloticus*) skin, bioactive peptides, tyrosinase inhibition, signaling pathway, molecular docking

## Abstract

The aim of this study was to investigate the anti-melanogenic effects and underlying mechanisms of PFRMY (Pro-Phe-Arg-Met-Tyr), a pentapeptide derived from tilapia skin (*Oreochromis niloticus*), using B16F10 murine melanoma cells. Treatment with PFRMY (1.0 mg/mL) significantly reduced intracellular melanin content and tyrosinase (TYR) activity by 39.55 ± 1.51% and 32.46 ± 1.31%, respectively. RT-PCR and Western blotting analyses revealed that PFRMY suppressed melanogenesis through the α-MSH/PKA/CREB signaling pathway. Notably, PFRMY reversed α-MSH-induced upregulation of key downstream factors including PKA, CREB, MITF, and TYR, while showing minimal effects on the protein expression of MC1R or α-MSH. Molecular docking further suggested that PFRMY binds to MC1R with higher affinity than α-MSH, potentially occupying the ligand-binding site and thereby interfering with downstream signaling. Collectively, these findings demonstrate that PFRMY effectively inhibits melanogenesis by competitively antagonizing the α-MSH/MC1R axis, highlighting its potential as a safe and efficacious ingredient for hyperpigmentation treatment and cosmetic applications.

## 1. Introduction

Normal pigmentation protects the skin from sunburn following UV radiation by mitigating intracellular DNA damage caused by oxidative stress. However, excessive melanin production can generate reactive oxygen species (ROS), which may in turn cause cellular damage [1]. Melanogenesis is regulated by several signaling pathways, including cAMP/PKA/CREB, MEK/ERK/MITF, p38 MAPK/CREB/MITF, and PI3K/AKT/GSK3β, among which the cAMP/PKA/CREB pathway is considered the most critical [2]. These hormonal factors often participate in multiple pathways and interact with one another. Among them, α-melanocortin (α-MSH) serves as the most important positive regulator [1]. By binding to the melanocortin 1 receptor (MC1R) on the surface of melanocyte membranes, α-MSH induces a conformational change in MC1R, thereby preventing binding by other ligands. This interaction promotes the dephosphorylation of ATP to cAMP, activating a cascade involving protein kinase A (PKA), cAMP-response element binding protein (CREB), microphthalmia-associated transcription factor (MITF), and tyrosinase (TYR). Regardless of which of these various signaling pathways is used, they end with the up-regulation of TYR activity because this is the sole step-limiting enzyme during melanin biosynthesis [3]. L-Tyrosine, a natural substrate of TYR, is catalyzed to dopaquinone and subsequently to eumelanin. TYR exhibits similar biological functions across various species, including humans, animals, plants, and microorganisms [4]. Thus, inhibiting TYR activity represents the most efficient strategy for suppressing melanogenesis to achieve skin-whitening effects.

Although the mechanisms underlying melanin production in animals are becoming increasingly clear, researchers continue to seek safer and more effective whitening agents, as existing options are not always satisfactory. Several whitening agents, such as kojic acid, 3-O-ethyl ascorbic acid, and hydroquinone, have been reported to possess potential carcinogenic and mutagenic properties [5]. Arbutin, one of the safest TYR inhibitors, can be hydrolyzed by skin microflora to release hydroquinone, thereby inducing cytotoxicity. Consequently, natural products have emerged as a key source of anti-melanogenic agents, with plant-derived compounds, particularly flavonoids, being more extensively studied than those from animal sources [6]. In recent years, bioactive peptides derived from food proteins have attracted increasing attention as safe and effective tyrosinase inhibitors [7,8]. Notably, fish processing by-products, such as skin and scales, have emerged as promising sources of bioactive peptides with antioxidant, anti-inflammatory, and anti-melanogenic activities [9]. Our previous research demonstrated that collagen hydrolysates from tilapia (*Oreochromis niloticus*) skin exhibit high melanin-scavenging activity [10]. A pentapeptide PFRMY and a hexapeptide RGFTGM were identified with good vitro TYR inhibitory activity (IC_50_ value was 0.43 mg/mL and 1.61 mg/mL respectively) and good copper chelating activity (15.70% and 14.44% when the concentration was 0.1 mg/mL, respectively) which is primarily considered relative to TYR inhibition. However, the underlying mechanisms by which these peptides exert their anti-melanogenic effects remain unclear. Recent studies have successfully elucidated the anti-melanogenic mechanisms of bioactive peptides through integrated approaches involving signaling pathway analysis and molecular docking [11,12]. In the present study, we used B16F10 murine melanoma cells to investigate the hypopigmentation effects and potential mechanisms of these two peptides. Our findings aim to provide a foundation for the development of whitening cosmetic ingredients and functional foods containing collagen-derived peptides.

## 2. Materials and Methods

### 2.1. Materials

Tilapia (*Oreochromis niloticus*) skin was obtained from Guangdong Baiwei Biotechnology (Foshan, China). B16F10 cells (CL-0319) and the exclusive medium (CM-0319) were purchased from Procell Life Science & Technology Co., Ltd. (Wuhan, China). Cell culture plates and centrifuge tubes were obtained from NEST Biotechnology Co., Ltd. (Wuxi, China). Mushroom tyrosinase (EC 1.14.18.1) and reduced glutathione (GSH) were provided from Sigma−Aldrich (St. Louis, MO, USA). PFRMY and RGFTGM were commercially synthesized by Yuanye Biotechnology Co., Ltd. (Shanghai, China) based on the sequences identified in our previous study [10]. The purities of PFRMY and RGFTGM were ≥97% and ≥95%, respectively. The synthetic peptides were further characterized by HPLC–ESI–MS. The detailed analytical conditions, including column specifications, gradient program, UV detection, and MS parameters, are provided in Supplementary Materials, Figure S1 and the characterization confirmed the purity and molecular identity of the synthetic peptides. Stock solutions of both peptides were prepared at a concentration of 10 mg/mL in sterile phosphate-buffered saline (PBS, pH 7.4), filtered through 0.22 μm membranes, and stored at −20 °C. Working solutions at indicated concentrations were freshly diluted from the stock solutions with cell culture medium prior to each experiment. α-MSH was obtained from R&D Systems (Minneapolis, MN, USA). L-dopa and α-arbutin were purchased from Macklin Co., Ltd. (Beijing, China). All the other chemicals were of analytical grade.

### 2.2. Cell Culture

B16F10 melanoma cells were cultured in the exclusive medium at 37 °C in a humidified 5% CO_2_ atmosphere. The cells were sub-cultured to logarithmic growth phase every 2 days and the different culture medium was used every day.

### 2.3. Cell Viability

The B16F10 cells (2 × 10^4^ cells/well) were cultured overnight in 96 well plate and then treated with different concentrations of samples (0.05–4.0 mg/mL) for 24, 48, and 72 h, which were dissolved in medium with/without α-MSH (100 nmol/L). The sample solution was changed every day, and cells cultured in medium alone served as the control. After the culture was completed, all the media were removed and cells were washed three times by phosphate buffer (PBS with pH 6.8). A cell viability assay was performed using Cell Counting Kit-8 (CCK-8, Solarbio Biotechnology Co., Ltd., Beijing, China) according to the manufacturers’ instructions. A total of 200 μL of medium and 10 μL of CCK-8 were added to the cell plate. The absorption was determined after incubation at 37 °C for 2 h and at 450 nm.

### 2.4. Melanin Content Assay

The assay was performed with minor modifications based on the method described by Teng et al. [13]. First, B16F10 cells were incubated in a 6-well plate for 24 h, then samples of various concentrations with/without 100 nmol/L α-MSH were added into the plate for 24 h, 48 h and 72 h. After trypsin digestion, cells were disrupted by 1.0 mol/L NaOH with 10% DMSO and heated at 80 °C for 30 min. The solution was centrifugated at 10,000× *g* (15 min, 4 °C) and sediments dissolving to 200 μL of PBS were used to measure the melanin content at 405 nm. The dissolved protein was determined by BCA Kit (Beyotime Biotechnology Co., Ltd., Shanghai, China) for normalization.

### 2.5. TYR Activity Assay

TYR activity was measured according to the methods of Park et al. [14] with slight modifications. First, B16F10 cells were incubated in a 6-well plate for 24 h, then samples of various concentrations with/without 100 nmol/L α-MSH were added into the plate for 24 h, 48 h and 72 h. After trypsin digestion, cells were disrupted by 200 μL of 10% TritonX-100 and saved at −20 °C for 30 min. The supernatant was centrifuged at 10,000× *g* (10 min, 4 °C), then 100 μL of 5 mmol/L L-dopa was added into the supernatant and cultured for 1 h at 37 °C. The formation rate of dopachrome was observed at 475 nm. The dissolved protein was determined by BCA Kit (Beyotime Biotechnology Co., Ltd., Shanghai, China) for normalization.

### 2.6. cAMP Content Assay

The cAMP content assay was measured by Mouse cAMP ELISA Kit (Mlbiao Technology Co., Ltd., Shanghai, China) according to the manufacturer’s instructions. The cells (1 × 10^7^ cells/mL) were treated with freeze–thawing five times to release intracellular components and the supernatant was collected by centrifugation at 5000× *g* (20 min, 25 °C) and normalized using BCA Protein Assay Kit (Beyotime Biotechnology Co., Ltd., Shanghai, China). The results for cells without PFRMY treatment provided a control of 1.0.

### 2.7. α-MSH Content Assay

The α-MSH content assay was evaluated by Guinea Pig Interleukin 1 (MSH) ELISA Kit (Meibiao Biotechnology Co., Ltd., Jiangsu, China) according to the manufacturer’s instructions. The B16F10 cells (2 × 10^5^ cells/well) were added with 200 μL of RIPA and then centrifugated at 5000× *g* (10 min, 4 °C). The supernatant of the culture medium was extracted to detect α-MSH content. The control group without PFRMY was 1.0.

### 2.8. RNA Isolation, cDNA Synthesis and RT-PCR

B16F10 cells were cultured with various concentrations of PFRMY for 48 h, then washed twice with PBS, and all RNA was extracted with TRIzol (Invitrogen, Carlsbad, CA, USA) according to the manufacturer’s guidance. Using a SYBR Green PCR Master Mix with oligo Dt (Jingge Technology, Hangzhou, China), all RNA was converted into cDNA, and then analyzed by RT-PCR by ViiA 7TM Real-Time PCR System (Applied Biosystems, Waltham, MA, USA). Glyceraldehyde 3-phosphate dehydrogenase (GAPDH) was used to normalize the relative gene expression using the ∆∆-CT-method. Cycling parameters were 95 °C for 10 min, 95 °C for 15 s, 40 cycles of 60 °C for 30 s, and 60 °C for 1 min. The primer sequence was as follows:

    Forward GAPDH, 5′-GGAGAAACCTGCCAAGTATGATGAC-3′    Reverse GAPDH, 3′-GAGACAACCTGGTCCTCAGTGTA-5′    Forward α-MSH, 5′-GCCGAGATTCTGCTACAGTCGC-3′    Reverse α-MSH, 3′-TTGCTCTCCGTGGTGAGGTCCT-5′    Forward MC1R, 5′-TCAGAGCCTTGGTGCCTGTA-3′    Reverse MC1R, 3′-GCAGGTTGCGGTTTTTGGTG-5′    Forward cAMP/Adcy1, 5′-GGTTGCTGGAGTGATCGGT-3′    Reverse cAMP/Adcy1, 3′-CGGTGGACTTCCTCAGTCA-5′    Forward MITF, 5′-CGACCTCTACAGCAACCAG-3′    Reverse MITF, 3′-GCTTCAGACTCTGTGGGGAA-5′    Forward CREB, 5′-GAGAACAGAGTGGCAGTGCTTGAA-3′    Reverse CREB, 3′-CCAGTCCATTCTCCACCGTAACAG-5′    Forward TYR, 5′-GTACAGGGATCGGCCAAC-3′    Reverse TYR, 3′-GGTGCATTGGCTTCTGGG-5′

### 2.9. Western Blotting Analysis

Western blotting analysis of MC1R, PKA, CREB, phosphor-CREB, MITF, phosphor-MITF and TYR was measured following the method described by Teng et al. [13]. The medium containing B16F10 cells with a density of 2 × 10^5^ cells/well was added with PFRMY at various concentrations, followed by 48 h incubation. A total of 1 mL of RIPA buffer was used to lyse cells washed with cold PBS on ice and centrifuged in 12,000× *g* (4 °C, 5 min). Total protein content from the supernatant was measured by the BCA method. The cell lytic product was separated by 10% SDS-PAGE (30–50 μg protein) and transferred to PVDF membrane (Rio-Rad, Hercules, CA, USA), and then blocked with 5% skim milk at room temperature for 2 h. The dilution ratio of different antibodies was as follows: PKA (1:2000), MC1R (1:2000), CREB (1:2000), phosphor–CREB (1:2000), MITF (1:2000), phosphor–MITF (1:2000) and TYR (1:2000). The PVDF membrane was incubated with secondary antibodies from Proteintech Co., Ltd. (Wuhan, China) at room temperature for 1 h. X-ray film (Yuehua, Beijing, China) was used to detect protein bands in the darkroom and the protein content was determined by ImageJ software (version 2.5.0, BioSpectrum 510 Imaging System Motorized Platform, Jena, Germany).

### 2.10. Molecular Docking Simulation

Pentapeptide PFRMY and α-MSH were used to simulate the possible binding poses with MC1R. A PDB format of MC1R (PDB_ID: 7F4D, chain E) and α-MSH (PDB_ID: 7F4D, chain F) was downloaded from RCSB Protein Data Bank (https://www.rcsb.org/structure/7F4D, accessed on 22 June 2024). The sequence of PFRMY was required to input the Chemdraw (version 19.0), aiming to obtain the 3D structure by Chem 3D (version 19.0), followed by minimizing the energy. For 7F4D, other unnecessary chains or ligands were removed, leaving only chain E as the MC1R and only chain F as α-MSH. All water molecules were removed and hydrogen atoms were added. The pentapeptide PFRMY and α-MSH were employed for docking simulations with MC1R within an all-inclusive grid box via AutoDock Vina (version 1.1.2). The best ligand binding pose was transferred to Discovery Studio (version 2020) to visualize the detailed interactions.

### 2.11. Statistical Analysis

An analysis of variance was carried out by using 18.0 SPSS statistical software, and the minimum significant difference (Duncan’ s) was based on one-way ANOVA. All the tests were carried out three times, and the test results were based on the average deviation.

## 3. Results and Discussion

### 3.1. Effect of Tilapia Skin Collagen Peptides on Cell Viability

**Figure 1:**
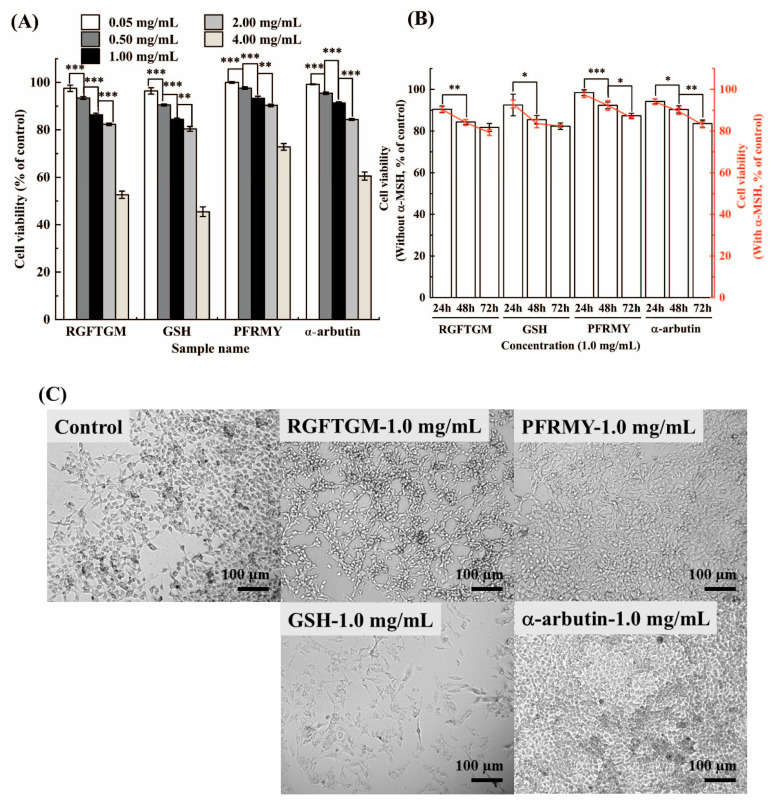
Effect of tilapia skin collagen peptides on B16F10 cell viability. Cell viability was assessed using the CCK-8 assay to evaluate the cytotoxicity of PFRMY, RGFTGM, α-arbutin, and GSH. Cells were precultured in complete medium for 24 h prior to treatment. (**A**) Concentration-dependent effects were observed after 48 h of treatment at concentrations ranging from 0.05 to 2.0 mg/mL. (**B**) Time-dependent effects were observed at a concentration of 1.0 mg/mL over 24, 48, and 72 h. (**C**) Representative microscopic images of B16F10 cells were observed after 48 h of treatment at 1.0 mg/mL. Cells cultured in complete medium for 48 h served as the control. Data are presented as mean ± SD (*n* = 3). (* *p* < 0.05, ** *p* < 0.01, and *** *p* < 0.001).

The active deposition of melanin after oxidative stress is attributed to skin suntan. A previous whitening agent, hydroquinone, is now limited in cosmetics because it can cause irreversible damage to melanocyte’s membrane lipids [15]. However, a glucosylated hydroquinone derivative α-arbutin exhibits much higher safety than hydroquinone. In addition, GSH is also regarded as a whitening agent because of its strong anti-oxidative capacity and biocompatibility. In our previous study, two peptides (PFRMY and RGFTGM) were identified from tilapia skin collagen hydrolysates [10]. For the present study, these peptides were commercially synthesized and their cell viability was compared with two popular whitening agents (α-arbutin and GSH) in the cosmetics industry. As shown in Figure 1A, cell viabilities were gradually inhibited due to the increasing concentration of samples and all cells showed above 80% viability under the sample concentration of 2.0 mg/mL, whereas 4.0 mg/mL samples experienced heavy damage. In addition, the cell viabilities decreased with the extension of culture time (Figure 1B). However, whether cells were exposed to α-MSH or not caused no significant difference, demonstrating that α-MSH had no obvious toxicity to cells. Surprisingly, α-arbutin demonstrated the highest cell viability, followed by PFRMY, while RGFTGM and GSH seemed to cause similar rates of cell death (Figure 1C), opposite to the results of Shen et al. [16]. This was possibly because they used β-arbutin instead of α-arbutin, as α-arbutin has been reported to exhibit approximately 10-fold higher tyrosinase inhibitory activity and superior safety compared to its β-anomer [17,18]. Different peptide sequences showed different levels of cytotoxicity, suggesting amino acid composition has a significant influence on peptide safety.

### 3.2. Anti-Melanogenic Effects on B16F10 Cells

**Figure 2:**
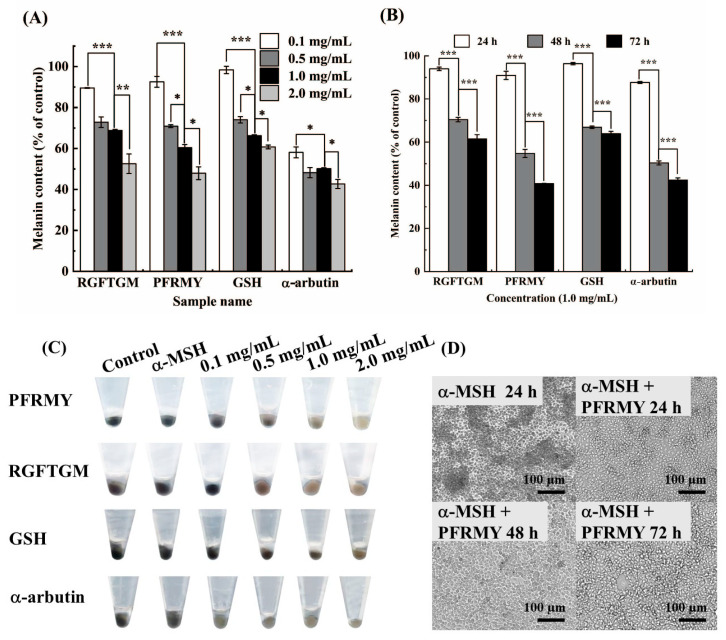
Anti-melanogenic effects of tilapia skin collagen peptides in B16F10 cells. Melanin content was measured spectrophotometrically and normalized to total protein to evaluate the inhibitory effects of PFRMY, RGFTGM, α-arbutin, and GSH. (**A**) Concentration-dependent inhibition after 48 h of treatment at concentrations of 0.1–2.0 mg/mL. (**B**) Time-dependent inhibition at a concentration of 1.0 mg/mL over 24, 48, and 72 h. (**C**) Representative images of melanin deposition in B16F10 cells after 48 h of treatment. (**D**) Representative microscopic images showing cell morphology and growth. α-MSH-treated cells served as the control. Data are presented as mean ± SD (*n* = 3). (* *p* < 0.05, ** *p* < 0.01, and *** *p* < 0.001).

The B16F10 murine melanoma cell strain is an easy and classic model for melanogenesis studies due to its high similarity to the human melanogenic mechanism [19]. Melanin synthesis is an important feature in the proliferation of B16F10 cells, which is mainly regulated by the TYR-related gene family. Studies have found that α-MSH can promote an increase in intracellular cAMP levels via its combination with MC1R, thus leading to an increase in the dendrite and melanin content of melanocytes [20]. Therefore, in this study we used α-MSH to establish a high-melanogenesis model, aiming to explore the melanin-scavenging effects of different samples, and the results are shown in Figure 2. As shown in Figure 2A, C, all samples experienced a significant reduction in melanin following treatment with above 0.5 mg/mL of sample solution. When the concentration was 1.0 mg/mL, PFRMY showed an inhibitory effect and melanin synthesis of 39.55 ± 1.51%, which was nearly equivalent to GSH at 2.0 mg/mL and α-arbutin at 0.1 mg/mL. The other peptide, RGFTGM, exhibited slightly better anti-melanogenetic effects than GSH. Although the addition of two tilapia skin collagen peptides did not lead to higher inhibitory activity than α-arbutin, they significantly reduced the melanin content compared with the control group, indicating the importance of tilapia peptides in the inhibition of melanin synthesis. Figure 2B showed that melanin synthesis significantly decreased over time, suggesting that the two tilapia peptides inhibit melanin synthesis in B16F10 cells. As shown in Figure 2D, B16F10 cells remarkably increase melanin production following treatment with α-MSH at 100 nmol/L compared to the blank group (100% of control), and PFRMY displayed a time-dependent inhibition on melanin production in B16F10 cells. These results demonstrated that PFRMY attenuated the melanogenesis that is mediated by adding α-MSH [13].

### 3.3. TYR Inhibitory Activity on B16F10 Cells

Melanin synthesis is accelerated by the oxidation of tyrosine or L-dopa, a process regulated by TYR activity in melanocytes [20]. Therefore, the inhibition of TYR plays a critical role in anti-melanogenesis. Consistent with the melanin content results, α-MSH treatment led to a 1.5-fold increase in TYR activity compared with the blank group.

**Figure 3:**
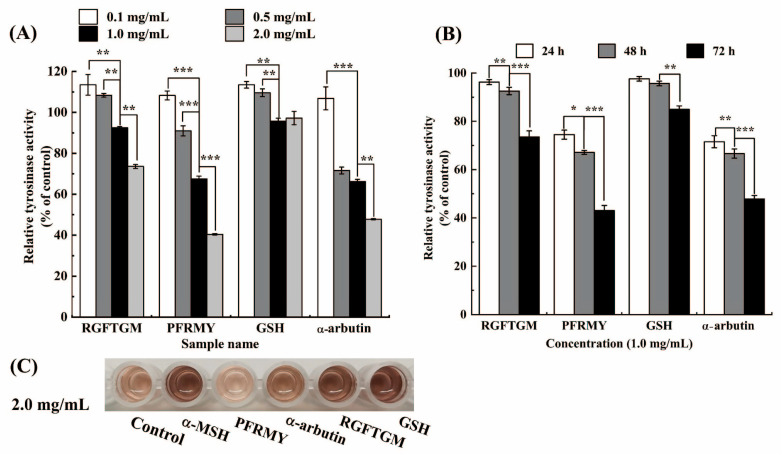
Inhibitory effects of tilapia skin collagen peptides on TYR activity in B16F10 cells. TYR activity was determined by measuring dopachrome formation from L-dopa and normalized to total protein. (**A**) Concentration-dependent inhibition after 48 h of treatment at concentrations of 0.1–2.0 mg/mL. (**B**) Time-dependent inhibition by PFRMY (1.0 mg/mL) over 24, 48, and 72 h. (**C**) Colorimetric changes in L-dopa solution following incubation with cell lysates from B16F10 cells treated with different samples. α-MSH-treated cells served as the control. Data are presented as mean ± SD (*n* = 3). (* *p* < 0.05, ** *p* < 0.01, and *** *p* < 0.001).

As shown in Figure 3A, all four samples significantly inhibited TYR catalytic activity. At concentrations below 0.5 mg/mL, α-arbutin exhibited a stronger inhibitory effect on TYR activity than the other three peptides, requiring a lower concentration to achieve comparable inhibition. However, at 2.0 mg/mL, PFRMY surpassed α-arbutin, achieving a 59.57 ± 0.34% reduction in TYR activity compared with 52.50 ± 0.25% for α-arbutin. RGFTGM ranked third, showing nearly 40% inhibition of TYR activity across concentrations from 0.5 to 2.0 mg/mL, whereas GSH exhibited only a modest inhibitory effect.

Previous studies have reported that arbutin acts as a substrate for oxy-tyrosinase [21], and that certain food-derived peptides, such as FTGML, can chelate the active center of TYR, thereby disrupting its catalytic function [22]. As demonstrated by molecular docking analysis in our previous study [10], the differential inhibitory activities against the TYR of PFRMY, RGFTGM, and GSH are likely attributable to their distinct binding modes within the TYR active site.

As shown in Figure 3B, PFRMY inhibited TYR activity in a time-dependent manner. This effect may result either from the direct inhibition of TYR catalytic activity or from the downregulation of upstream genes that regulate TYR expression, a possibility that warrants further investigation.

### 3.4. TYR-Related Gene Pathways in B16F10 Cells

**Figure 4:**
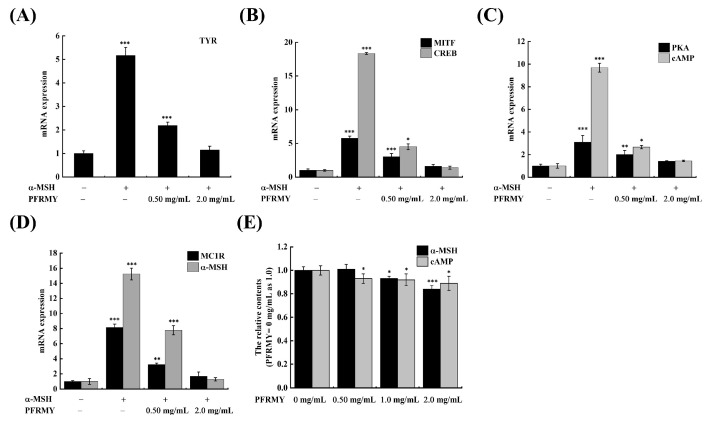

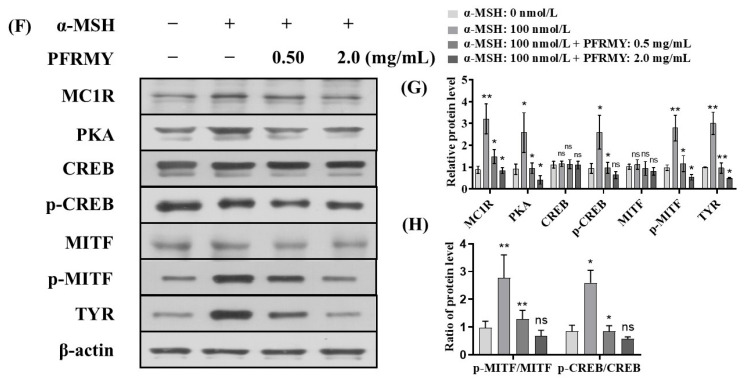
Effects of PFRMY on the α-MSH/PKA/CREB signaling pathway in B16F10 cells. B16F10 cells were treated with PFRMY (0.5, 1.0, or 2.0 mg/mL) in the presence of 100 nmol/L α-MSH for 48 h. (**A**) Relative mRNA expression levels of TYR, MITF, CREB, PKA, cAMP, MC1R, and α-MSH, determined by RT-PCR. (**B**) Relative mRNA expression of MITF and CREB. (**C**) Relative mRNA expression of PKA and cAMP. (**D**) Relative mRNA expression of MC1R and α-MSH. (**E**) Relative protein levels of α-MSH and cAMP, measured by ELISA. (**F**) Protein expression levels of melanogenic proteins (MC1R, PKA, CREB, p-CREB, MITF, p-MITF, TYR) determined by Western blotting, with β-actin as the internal control. Cells cultured in medium alone served as the control. (**G**) Relative protein level compared with control group. (**H**) p-MITF/MITF ratio and p-CREB/CREB ratio of relative protein levels compared with the control group. Data are presented as mean ± SD (*n* = 3). (* *p* < 0.05, ** *p* < 0.01, and *** *p* < 0.001).

As shown in Figure 4, the transcription levels of all key genes were markedly increased upon α-MSH treatment, indicating that α-MSH serves as an upstream initiator of melanogenesis. TYR, the rate-limiting enzyme in melanin biosynthesis, is also a downstream factor that is directly regulated by other genes [6]. As shown in Figure 4A, F, both *TYR* mRNA expression and TYR protein level exhibited a downward trend with increasing concentrations of PFRMY. These results confirm that PFRMY inhibits melanogenesis at least in part through the suppression of the TYR signaling pathway.

To identify the key upstream regulators of TYR within this pathway, we further examined the expression of transcription factors involved in melanogenesis. MITF, a critical transcription factor controlling melanogenic enzymes, activates *TYR* expression by binding to M-box or E-box consensus motifs [23]. MITF itself receives signals from CREB, whose phosphorylation is a key event regulating *MITF* transcription [24]. As can be seen from Figure 4B, the mRNA expression of *CREB* and *MITF* genes decreased significantly during PFRMY treatment, but their protein expression was different. After PFRMY treatment, the expression of CREB did not change significantly, while the expression of p-CREB tended to decrease. The protein level of MITF showed no significant change with increasing PFRMY concentration, and p-MITF showed a comparable dose-dependent reduction (Figure 4F,G). In addition, the ratios of p-MITF/MITF and p-CREB/CREB increased with α-MSH treatment, and then were inhibited by PFRMY in a dose-dependent manner (Figure 4H), indicating PFRMY interruption of this signaling pathway. In agreement with our findings, a previous study reported that there was a positive correlation between the action concentration of cefotaxime sodium and the protein inhibition level of MITF and p-CREB [22]. The results reflected that PFRMY inhibited melanogenesis through the *CREB*/*MITF*-mediated downregulation of *TYR* in B16F10 cells. In melanocytes, the increased content of *cAMP* indicates the initiation of the melanin gene pathway because it stimulates the PKA translocation responsible for the phosphorylation of CREB and MITF. As shown in Figure 4C, PFRMY treatment reversed the α-MSH-induced increase in cAMP to near-normal levels, and PKA as well. At the same time, PFRMY could also inhibit the expression of PKA in a concentration-dependent manner. The results shown in Figure 4E further verify PFRMY inhibited cAMP-mediated melanin production.

Upon UV exposure, melanotropic hormones such as α-MSH bind to MC1R, leading to adenylyl cyclase activation, which catalyzes the conversion of ATP to cAMP and subsequently triggers eumelanin biosynthesis [25]. As a transmembrane protein, MC1R adopts a U-shaped conformation in its extracellular domain. α-MSH is a 13-residue peptide hormone that exhibits a nearly identical conformation to the MC1R ligand [26]. Antagonistic binding to MC1R would block its activation, thereby inhibiting the PKA/CREB/MITF signaling pathway. Competitive binding to MC1R has been reported as a common mechanism for several natural anti-melanogenic agents, including peptides and small molecules [27]. As shown in Figure 4D,F, PFRMY treatment induced a sharp decrease in *MC1R* mRNA expression, whereas its protein level remained largely unchanged. In addition, Figure 4E showed a slight decrease in α-MSH content in B16F10 cells following PFRMY treatment. Based on these observations, we speculated that PFRMY might affect MC1R function and melanin production through competitive binding with MC1R. To explore this possibility, the interactions of PFRMY and α-MSH with MC1R were investigated using molecular docking simulations.

### 3.5. Molecular Docking

**Figure 5:**
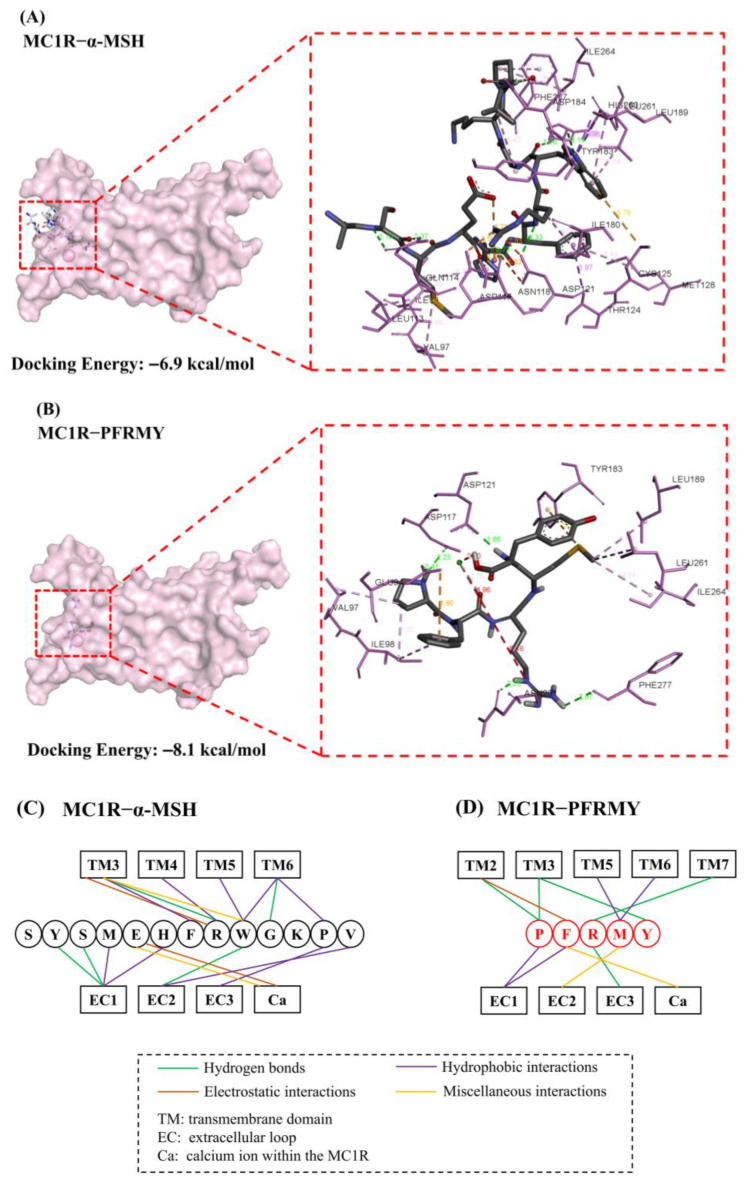
Molecular docking analysis of PFRMY and α-MSH binding to MC1R. The binding modes of PFRMY and α-MSH with MC1R were simulated using AutoDock Vina based on the crystal structure of MC1R (PDB: 7F4D, chain E). (**A**) Predicted binding pose of α-MSH within the binding pocket of MC1R. (**B**) Predicted binding pose of PFRMY. (**C**) Two-dimensional interaction diagram showing hydrogen bonds and hydrophobic interactions between α-MSH and MC1R. (**D**) Two-dimensional interaction diagram between PFRMY and MC1R.

Molecular docking has been widely employed to investigate the binding modes of MC1R with various ligands, providing valuable insights into the structural basis of receptor activation and inhibition [28,29]. Through the molecular docking of PFRMY/α-MSH and MC1R (Figure 5A,B), we found that PFRMY had the lowest docking energy of −8.1 kcal/mol when α-MSH was −6.9 kcal/mol, indicating a more competitive binding to MC1R. Among them, hydrogen bonding and hydrophobic forces played a leading role. PFRMY formed hydrogen bonds with Glu94, Asp117, Asp121, Phe277 and Asn281 and hydrophobic interactions with Val97, Ile98, Leu189, Leu261 and Ile264. α-MSH utilized Gln114, Tyr183, Asp121 and His260 to form hydrogen bonds and interacted with Val97, Ile98, Leu113, Thr124, Met128, Ile180, Tyr183, Leu189, Leu261, Ile264 and Phe277 through hydrophobic forces. Both peptides bond to calcium ions (Ca^2+^), proving that they insert the hydrophobic binding pocket of MC1R. As shown in Figure 5C, D, His-Phe-Arg-Trp (HFRW) of α-MSH provided the major contacts for binding to MC1R with transmembrane domain 3–6 (TM3–TM6) while PFRM played a similar role in PFRMY.

Previous studies have clarified that there is a U-shape conformation formed by extracellular loops of MC1R and a Ca^2+^ binding pocket formed by Ala94, Asp117 and Asp121 at the center of the conformation [30]. As the most important agonist of MC1R, α-MSH penetrates deep into the core of the U-shape conformation through the highly conserved HFRW motif [31]. From the results of the docking simulation, we found PFRMY bound to Asp117 (2.23 Å) and Asp121 (1.86 Å) of MC1R through hydrogen forces when α-MSH exhibited a longer distance between two residues (2.58 Å and 2.83 Å, respectively). Furthermore, residue Phe of PFRMY formed more different interactions with MC1R than α-MSH, suggesting that PFRMY had higher affinity for the binding domain of MC1R because Phe played a key role in the binding between G protein-coupled receptors and melanocortin receptors. The cryo-electron microscopy structure of the MC1R-α-MSH complex revealed that short extracellular loops EC2 and EC3 were responsible for holding relative peptide hormones and TM6 was critical for MC1R activation [30]. In addition, TM4−TM6 consist of a series of aromatic residues such as Tyr and Phe that form extensive hydrophobic interactions with PFRMY. Collectively speaking, pentapeptide PFRMY presented almost all of the previously reported important interactions and it verified our hypothesis that PFRMY was more competitive for binding to MC1R than α-MSH.

## 4. Conclusions

Our results revealed that the pentapeptide PFRMY, obtained from tilapia skin collagen hydrolysate (*Oreochromis niloticus*), exhibited good safety and an excellent melanin scavenging ability in B16F10 cells. Despite inhibiting the activity of TYR, PFRMY was also able to suppress the expression of related genes upstream of TYR, such as PKA, MC1R, CREB and MITF enhanced by α-MSH. It was proposed that PFRMY competitively binds to MC1R against α-MSH, thereby regulating the PKA/CREB/MITF signaling pathway. In addition, PFRMY exhibited higher safety and improved melanin scavenging activity compared to α-arbutin, suggesting its potential as a promising candidate for the treatment of hyperpigmentation disorders.

Although bioactive peptides offer considerable therapeutic potential, their application in vivo is often limited by rapid enzymatic degradation and poor bioavailability [32]. Emerging strategies such as encapsulation in nanocarriers, chemical modification (e.g., cyclization or conjugation with penetration enhancers), and formulation with protease inhibitors have shown promise in improving the stability and delivery of peptide-based agents. Recent in vivo studies have demonstrated the potential of collagen-derived peptides in ameliorating hyperpigmentation in animal models [33], supporting the need for further translational research. More studies are warranted to evaluate its bioavailability and efficacy.

## Data Availability

The original contributions presented in the study are included in the article, further inquiries can be directed to the corresponding author.

## Supplementary Materials

The following supporting information can be downloaded at: https://www.mdpi.com/article/10.3390/foods15081378/s1, Figure S1. (A) LC-MS/MS data for PFRMY. (B) LC-MS/MS data for RGFTGM.

## Abbreviations

The following abbreviations are used in this manuscript:

PFRMY	Proline-phenylalanine-arginine-methionine-tyrosine
TYR	Tyrosinase
PKA	Protein kinase A
CREB	cAMP-response element binding protein
MITF	Microphthalmia-associated transcription factor
MC1R	Melanocortin 1 receptor
α-MSH	α-Melanocortin
